# Chondrosarcomatous Differentiation in a Large Malignant Melanoma of the Scalp

**DOI:** 10.1155/2016/1063569

**Published:** 2016-12-18

**Authors:** Colin S. Alderson, Helen E. Douglas, Rebecca Rollett, Bipin Mathew, Paolo Matteucci

**Affiliations:** ^1^Leeds Teaching Hospitals Trust, Leeds, UK; ^2^Castle Hill Hospital, East Yorkshire, UK

## Abstract

*Background*. Divergent differentiation in malignant melanoma is a rare phenomenon, which can lead to delayed diagnosis or misdiagnosis, impacting upon patient treatment and outcome, as well as the understanding of tumour behaviour.* Case*. We present the case of a large long-standing tumour on the scalp of a 72-year-old female patient, which when excised and examined histologically was revealed to be a nodular malignant melanoma displaying chondrosarcomatous differentiation. Foci suggestive of lentigo maligna were also present. Rapid metastatic spread of the tumour was observed shortly after the primary resection.* Discussion*. To our knowledge, this is the first reported case in the literature of chondrosarcomatous differentiation in a lentigo maligna melanoma. The clinical and histopathological details and images of this case are presented alongside a discussion regarding such tumours and patterns of similar tumour behaviour.

## 1. Introduction

Cutaneous malignant melanoma is the deadliest primary skin cancer. The incidence and mortality figures for malignant melanoma have continued to rise in the UK, US, Australia, New Zealand, and Western Europe over the past decades [1, 2]. Atypical forms of cutaneous malignant melanoma can prove diagnostically difficult for both clinicians and histopathologists. Divergent differentiation in malignant melanoma is a rare phenomenon, which can lead to delayed diagnosis or misdiagnosis, impacting upon patient treatment and outcome and the understanding of tumour behaviour.

## 2. Clinical Case

A 72-year-old lady was referred to plastic surgery outpatient services due to a long-standing growth on her scalp; this was reported to have been present and slowly enlarging for 15 years [3]. She reported no symptoms from the lesion but explained it had more rapidly increased in size over the past 2 years.

The patient was fit and well, took propranolol for anxiety, but no other medications, and had no personal or family history of skin pathology.

On examination there was a large, fixed spherical mass on the left parietal-posterior scalp with central ulceration and multiple subcutaneous satellite nodules abutting the left pinna.

At initial assessment an incision biopsy was performed, which revealed a malignant melanoma at least 2.65 mm thick, with a mitotic count of 2/mm^2^.

A CT scan of the head, neck, thorax, and abdomen reported a large left parietal soft tissue mass with no bony involvement, normal intracranial appearances, and some small left postauricular lymph nodes, but no evidence of distant metastasis.

**Figure 1:**
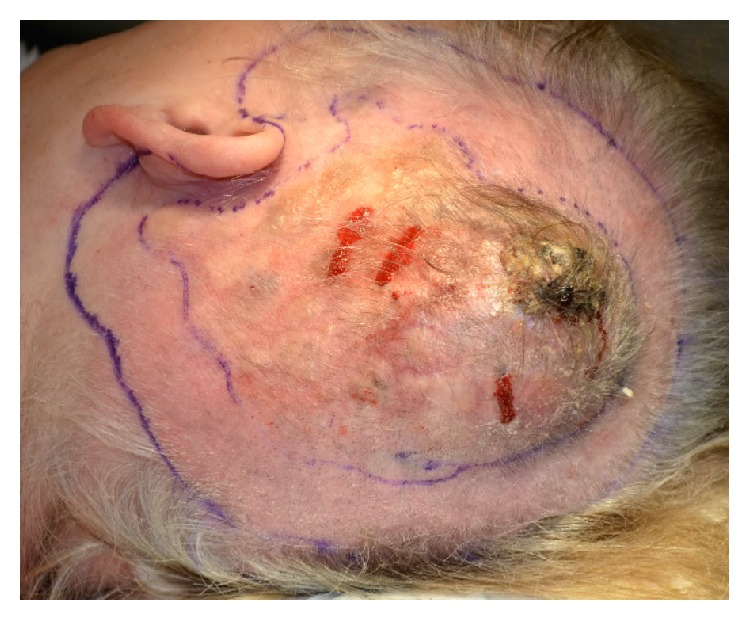
Primary tumour at time of resection.

The patient was discussed at the head and neck cancer multidisciplinary team meeting and underwent resection of the lesion, with 3 cm margins including the pericranium, level II-V neck dissection and reconstruction with a free latissimus dorsi muscle flap and covering split skin graft (see Figure 1).

**Figure 2:**
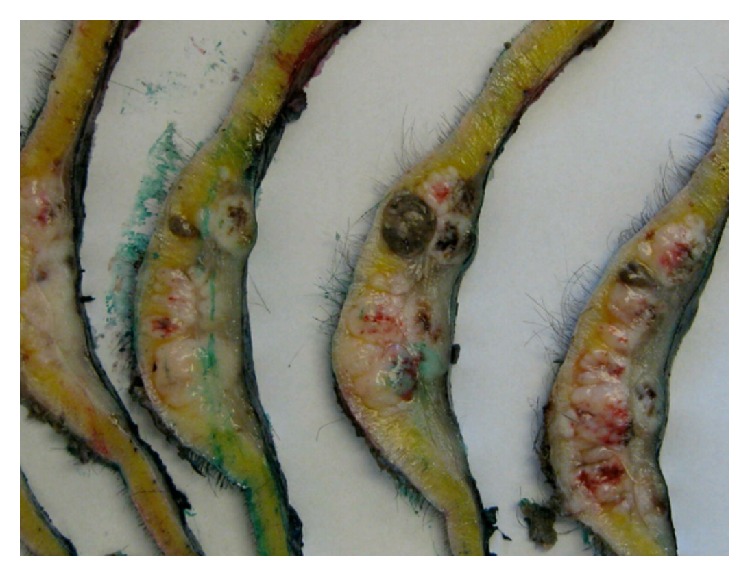
Macroscopic tumour histopathology showing in-transit disease.

Macroscopic examination showed a specimen which was 175 mm × 165 mm and 6 mm thick, with dermal nodules more than 1 cm from the main lesion indicating in-transit metastases (see Figure 2).

**Figure 3:**
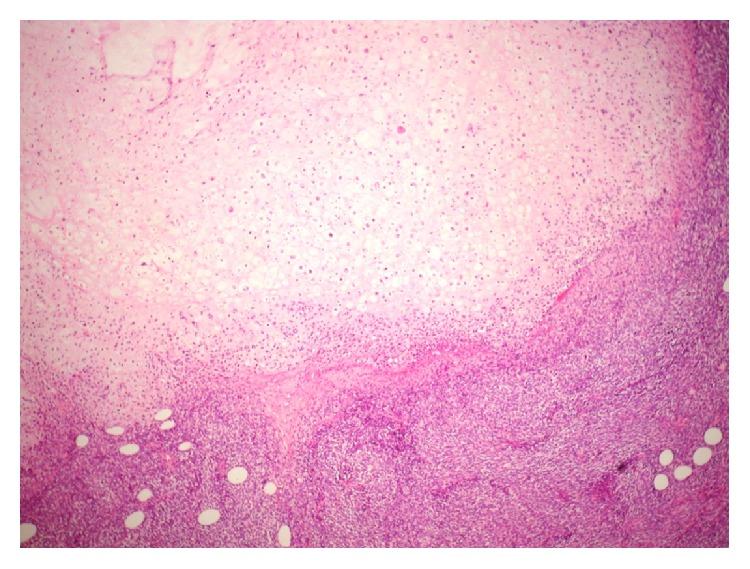
Microscopic histopathology of the tumour showing chondrosarcomatous transformation (×100 magnification).

**Figure 4:**
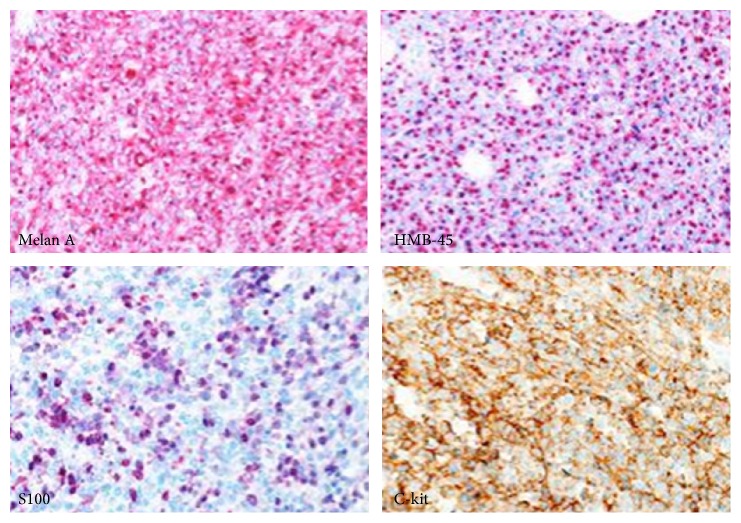
Microscopic histopathology of tumour with Melan A, HMB-45, and C-kit staining (×200 magnification).

Microscopic examination revealed a nodular malignant melanoma 22.1 mm thick with perineural invasion and a mitotic count of 17/mm^2^. There existed a heterogeneous morphology characterised by spindle cell neurofibroma-like areas, small cell areas, epithelioid cell areas, and focal chondrosarcomatous divergent differentiation (see Figure 3) with a focal lentigo maligna-like in situ component. The tumour showed patchy but strong staining with Melan A, HMB-45, and C-kit (see Figure 4). All lymph nodes were negative. On mutational analysis the tumour did not show BRAF V600E or C-kit mutation.

## 3. Discussion

Divergent differentiation in melanoma is very rare and to our knowledge chondrosarcomatous differentiation in a lentigo maligna melanoma that is a melanoma in chronic sun-damaged skin, has not been previously reported in the literature.

Banerjee and Eyden (2008) in their review of divergent differentiation in melanomas highlight that osteocartilaginous differentiation in melanomas may lead to misdiagnosis of osteosarcoma or chondrosarcoma, which can delay correct diagnosis and treatment [4]. A Medline review of the literature for sarcomatous differentiation in lentigo maligna revealed two review articles [5, 6] and a small case series of desmoplastic malignant melanomas [7]. None of these articles report chondrosarcomatous differentiation in lentigo maligna melanoma.

Mutation analysis showed no evidence of C-kit or BRAF 600E mutation. KIT is an important oncogene in melanoma. In a study of 102 primary melanomas by Curtin et al., mutations and/or copy number increases of KIT were present in 39% of mucosal and 36% of acral melanomas, in 28% of melanomas occurring in chronically sun-damaged skin as evidenced by dermal elastosis, but not in any (0%) melanomas on skin without chronic sun damage [8]. BRAF is commonly mutated in melanomas on intermittently sun-exposed skin while those on mucosal membranes, acral skin (soles, palms, and nail bed), and skin with chronic sun-induced damage only rarely show mutations [9–11].

## 4. Conclusions

This case outlines an unusual type of tumour differentiation in a long-standing indolent tumour that had recently become more aggressive. The clinical implications of such unusual tumour differentiation are not known; however correct identification of the cellular nature of such tumours and their immunohistochemical status is vital to improve our collective knowledge and to help treatment planning.

This patient developed multiple distant metastases in a matter of weeks following her tumour resection and reconstruction. This phenomenon of rapid disease progression following primary tumour resection in long-standing melanomas has been observed in several patients by our group. While this pattern is purely observational, our group have raised the question of whether, in these cases, some form of immunity from widespread metastases is provided while the primary tumour remains intact. We are currently examining our series to try to gather more information regarding this observation.

## References

[1] Forsea A. M., Del Marmol V., De Vries E., Bailey E. E., Geller A. C. (2012). Melanoma incidence and mortality in Europe: new estimates, persistent disparities. *British Journal of Dermatology*.

[2] Sneyd M. J., Cox B. (2013). A comparison of trends in melanoma mortality in New Zealand and Australia: the two countries with the highest melanoma incidence and mortality in the world. *BMC Cancer*.

[3] Douglas H., Rollett R., Mathew B., Matteucci P. Chondrosarcomatous differentiation in a large malignant melanoma of the scalp.

[4] Banerjee S. S., Eyden B. (2008). Divergent differentiation in malignant melanomas: a review. *Histopathology*.

[5] Lopansri S., Mihm M. C. (1979). Clinical and pathological correlation of malignant melanoma. *Journal of Cutaneous Pathology*.

[6] Barnhill R. L., Mihm M. C. (1993). The histopathology of cutaneous malignant melanoma. *Seminars in Diagnostic Pathology*.

[7] Riccioni L., Di Tommaso L., Collina G. (1999). Actin-rich desmoplastic malignant melanoma: report of three cases. *The American Journal of Dermatopathology*.

[8] Curtin J. A., Busam K., Pinkel D., Bastian B. C. (2006). Somatic activation of KIT in distinct subtypes of melanoma. *Journal of Clinical Oncology*.

[9] Handolias D., Hamilton A. L., Salemi R. (2010). Clinical responses observed with imatinib or sorafenib in melanoma patients expressing mutations in KIT. *British Journal of Cancer*.

[10] Gray-Schopfer V., Wellbrock C., Marais R. (2007). Melanoma biology and new targeted therapy. *Nature*.

[11] Chin L., Garraway L. A., Fisher D. E. (2006). Malignant melanoma: genetics and therapeutics in the genomic era. *Genes and Development*.

